# Visual and Refractive Outcomes following Bilateral Implantation of Extended Range of Vision Intraocular Lens with Micromonovision

**DOI:** 10.1155/2018/7321794

**Published:** 2018-02-06

**Authors:** Sri Ganesh, Sheetal Brar, Archana Pawar, Kirti J. Relekar

**Affiliations:** Nethradhama Superspeciality Eye Hospital, Bengaluru, Karnataka, India

## Abstract

**Purpose:**

To evaluate the outcomes following bilateral ERV intraocular lens implantation with micromonovision.

**Methods:**

25 subjects underwent bilateral Tecnis Symfony IOL implantation with micromonovision. The dominant eye was targeted for emmetropia and the nondominant eye for myopia of −0.75 D. Uncorrected and corrected distance (UDVA, CDVA), intermediate (UIVA, CIVA), and near visual acuity (UNVA, DCNVA); reading performance; defocus curve; and contrast sensitivity were studied. Follow-ups were conducted at 1 week and 1 and 6 months postoperatively.

**Results:**

At 6 months postoperatively, the mean binocular UDVA, CDVA, UNVA, and DCNVA were −0.036 ± 0.09, −0.108 ± 0.07, 0.152 ± 0.11, and 0.216 ± 0.10 logMAR, respectively. Binocular UIVA and DCIVA were 0.048 ± 0.09 and 0.104 ± 0.08 logMAR, respectively, at 60 cm and −0.044 ± 0.09 and 0.012 ± 0.09 logMAR, respectively, at 80 cm. All patients had ≥0.2 logMAR UDVA and UNVA. Reading acuity and reading speeds showed improvement over time. Between defocus range of −2.50 and +1.00 D, the visual acuity remained ≥0.2 logMAR. Contrast sensitivity scores were within the normal range. 4 patients used reading glasses for very fine print.

**Conclusion:**

Bilateral ERV IOL implantation leads to excellent outcomes for far and intermediate vision, satisfactory outcomes for near vision, and good tolerance to micromonovision at the end of the 6 months. This trial is registered with CTRI/2015/10/006246.

## 1. Introduction

Multifocal IOLs were reported to provide higher patient satisfaction due to better results for near and intermediate vision and a greater depth of focus, due to which they appear to have higher spectacle independence and patient satisfaction than monofocal IOLs [1, 2]. The early-generation multifocal IOLs, however, were shown to have noteworthy limitations, such as inferior contrast sensitivity and increased higher-order aberrations as compared with monofocal IOLs [3]. It is known that contrast sensitivity reduces with the progression of age due to spherical aberrations [4, 5].

Since the spherical IOLs do not address spherical aberration as do aspheric IOLs, the latter has been shown to produce comparatively better functional vision outcomes [6, 7]. Correction of ocular chromatic aberrations, in addition, also demonstrated improvement in the overall optical quality following cataract surgery by reducing blur and contrast vision [8–10].

The recently introduced Tecnis Symfony IOL (Johnson & Johnson, New Jersey, USA) is based on this concept of correction of chromatic aberration through a proprietary achromatic technology. In addition, the IOL is claimed to extend the range of vision by virtue of its novel, diffractive step-like optical profile [11].

However, it is speculated that the elongated focus provided by this lens results in better outcomes for uncorrected far and intermediate vision compared to near vision. Hence, in order to achieve satisfactory outcomes for near vision, the time-tested concept of mini-/micromonovision following the bilateral implantation of this lens may be attempted [12].

The current study was conducted to evaluate the visual, refractive, and contrast sensitivity; reading performance; and patient satisfaction outcomes with this new extended range of vision intraocular lens (ERV IOL) and confirm the benefits of micromonovision, if any, in a 6-month prospective, clinical trial.

## 2. Materials and Methods

This prospective, single-centre study included 50 eyes from 25 patients undergoing bilateral cataract surgery with implantation of the Tecnis Symfony IOL (Johnson & Johnson, New Jersey, USA) which is an extended range of vision IOL.

The study was approved by the hospital ethics committee of Nethradhama Superspeciality Eye Hospital and conducted in accordance with the principles of the Declaration of Helsinki. All patients provided written informed consent.

Inclusion criteria were healthy eyes besides senile cataract; corneal astigmatism equal to or less than 1.00 dioptres (D); IOL powers between +10.00 D and +32.00 D, in the capsular bag IOL implantation; and ability to read English language fluently.

Exclusion criteria were patients with irregular astigmatism, corneal dystrophy, pupillary abnormalities, history of glaucoma or intraocular inflammation, macular disease or retinopathy, neuroophthalmic diseases, and intraoperative or postoperative complications.

### 2.1. Preoperative Assessment and IOL Power Calculation

Preoperatively, all patients underwent complete ophthalmologic examination including manifest refraction, slit-lamp biomicroscopy, noncontact tonometry, and dilated fundus examination.

Axial length was measured with the IOL Master 500 (Carl Zeiss Meditec, Jena, Germany), and IOL power was calculated using the SRK-T formula. Dominance of the eye was tested using the shooting/hole in a card test. In all patients, the dominant eye was targeted for emmetropia and the nondominant eye was targeted at a myopia of −0.75 D.

Postoperative follow-up examinations were performed at 1 day, 1 week, 1 month, and 6 months after surgery. Slit-lamp examination was performed on the day after surgery. The following tests were performed at all postoperative visits from the first week: measurement of binocular uncorrected (UDVA) and corrected distance visual acuity (CDVA), binocular uncorrected (UNVA) and distance-corrected near visual acuity (DCNVA) at 40 cm, and binocular uncorrected (UIVA) and distance-corrected intermediate visual acuity (DCIVA) at 60 cm using ETDRS charts (Precision Vision, La Sella, IL, USA); binocular mesopic contrast sensitivity testing (F.A.C.T. Stereo Optical Co. Inc., Chicago) with distance correction; and measurement of uncorrected and distance-corrected defocus curves. Different levels of defocus were introduced in 0.50 D steps from +2.50 to −2.50 D.

Reading performance was evaluated using the Salzburg reading desk (SRD) (University Eye Clinic, Paracelsus Medical University of Salzburg, Austria) which provides for controlled reading distance and automated calculation of the reading speed and logarithmic reading acuity. At each postoperative follow-up from one week onwards, uncorrected and distance-corrected reading acuity (UCRA and DCRA) and uncorrected and distance-corrected reading speeds (UCRS and DCRS) (speed associated with maximum reading acuity) with a minimum reading speed of 80 words per minute (wpm), representing the lower limit for recreational sense-capturing reading, were evaluated [13, 14]. All measurements of reading performance were performed with and without distance correction.

At the last follow-up visit, a subjective questionnaire was obtained from all patients regarding dysphotopsia symptoms and spectacle independence for various activities.

### 2.2. Surgical Technique

All surgeries were performed by a single experienced refractive surgeon (S.G.), using a standard phacoemulsification technique under topical anesthesia. The UNFOLDER Platinum 1 Series Screw-Style Inserter (Johnson & Johnson, New Jersey, USA) was used to inject the IOL through a 2.8 mm temporal clear corneal incision. Postoperative topical therapy included topical prednisolone (1%, Pred Forte, Allergan), moxifloxacin (0.5%, Vigamox, Alcon), and nepafenac (0.1%, Nevanac, Alcon).

### 2.3. Statistical Analysis

SPSS software for Windows version 17.0.0 (IBM Corp., Armonk, NY) was used for statistical analysis. Normality of data samples was evaluated by the Kolmogorov–Smirnov test. When parametric analysis was possible, Student's *t*-test for paired data was used, whereas the Mann–Whitney test was applied to assess the significance of such differences when parametric analysis was not possible. All values were expressed as mean ± standard deviation (SD). A *p* value of 0.05 or less was considered statistically significant.

## 3. Results

A total of 25 patients with a mean age of 60.76 ± 10.74 years, undergoing bilateral Tecnis Symfony IOL implantation, were recruited in the study. Since micromonovision was performed, the postoperative visual outcomes and reading performance were evaluated binocularly (Table 1).

## 4. Distance Visual Acuity and Refraction

**Figure 1:**
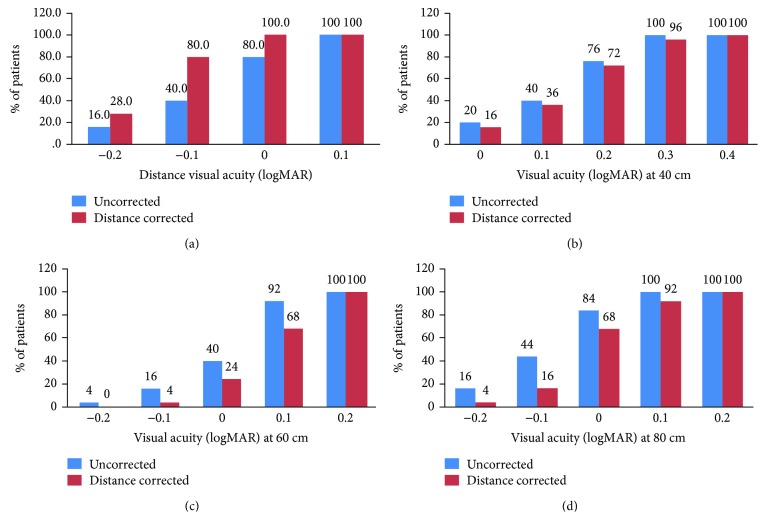
Cumulative binocular visual outcomes for (a) distance, (b) near, (c) intermediate at 60 cm and (d) intermediate at 80 cm at 6 months postoperatively.

Figure 1 shows the cumulative percentage of eyes with logMAR UDVA and CDVA, 6 months after the surgery. 80% (20/25) of the patients had binocular UDVA of ≤0 logMAR. The UDVA was ≤0.1 logMAR in all 25 (100%) patients. All eyes achieved ≤0 logMAR CDVA. The binocular CDVA however was significantly better compared to UDVA (*p* = 0.007), as would be expected from the intentional targeting of −0.75 D in the nondominant eye. Postoperatively, the UDVA and CDVA did not change significantly between 1 week, 1 month, and 6 months (*p* value > 0.05 for all postop visits compared to 1 week) (Table 2).

At 6 months, the binocular UNVA at 40 cm was ≤0.3 logMAR in all 25 (100%) patients with a mean UNVA of 0.157 ± 0.11 (Figure 1). There was a significant improvement in binocular UNVA at the last follow-up compared to one week (*p* = 0.05) which was significantly better compared to binocular DCNVA (*p* = 0.05), as expected from the intentional targeting of −0.75 D in the nondominant eye, demonstrating the functional benefit of micromonovision.

In the intermediate range, the UCIVA at 80 cm measured with ETDRS charts was significantly better compared to that at 60 cm at all postoperative visits (*p* value < 0.05) (Table 3). At 6 months, the UCIVA showed a statistically significant improvement at both distances compared to 1 week (*p* values = 0.02 for 60 cm and 0.05 for 80 cm), with the UCIVA being significantly better compared to DCIVA for both distances (*p* < 0.05) (Table 2), again demonstrating the functional benefit of micromonovision.

### 4.1. Reading Acuity and Reading speeds

Binocular intermediate UCRA, DCRA, UCRS, and DCRS were comparable at both 60 cm and 80 cm, with no statistically significant differences between the values of these parameters at all postoperative visits (Table 3).

Binocular UCRA, DCRA, UCRS, and DCRS showed improvement from one week to 6 months postoperatively for all distances, with the improvement being significantly better for UCRA at 40 cm at the last visit (Table 4).

## 5. Stability of SE Refraction Over Time

**Figure 2:**
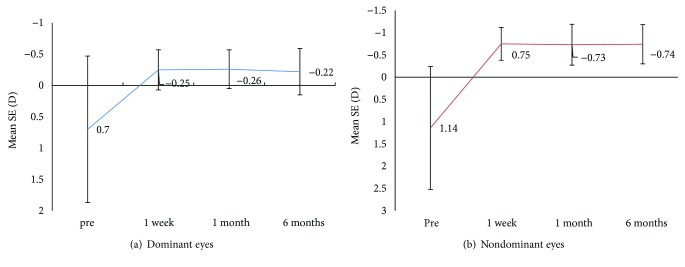
Stability of spherical equivalent (SE) correction for (a) dominant eyes and (b) nondominant eyes over time.

Postoperatively at one week, for both dominant and nondominant eyes, there was a statistically significant reduction in SE after surgery compared to preoperative SE. (*p* < 0.05) (Table 5). The values of mean SE in the dominant and nondominant eyes at 1 week were −0.25 ± 0.32 D and −0.75 ± 0.37 D, respectively, which slightly reduced to −0.22 ± 0.37 D and −0.74 ± 0.44 D, respectively, at the end of 6 months. However, there were no statistically significant differences in spherical equivalent between 1 week and 6 months postoperatively (*p* > 0.05) (Figure 2). 92% of the dominant eyes were within ±0.05 D of SE correction at the end of 6 months.

## 6. Defocus Curves

**Figure 3:**
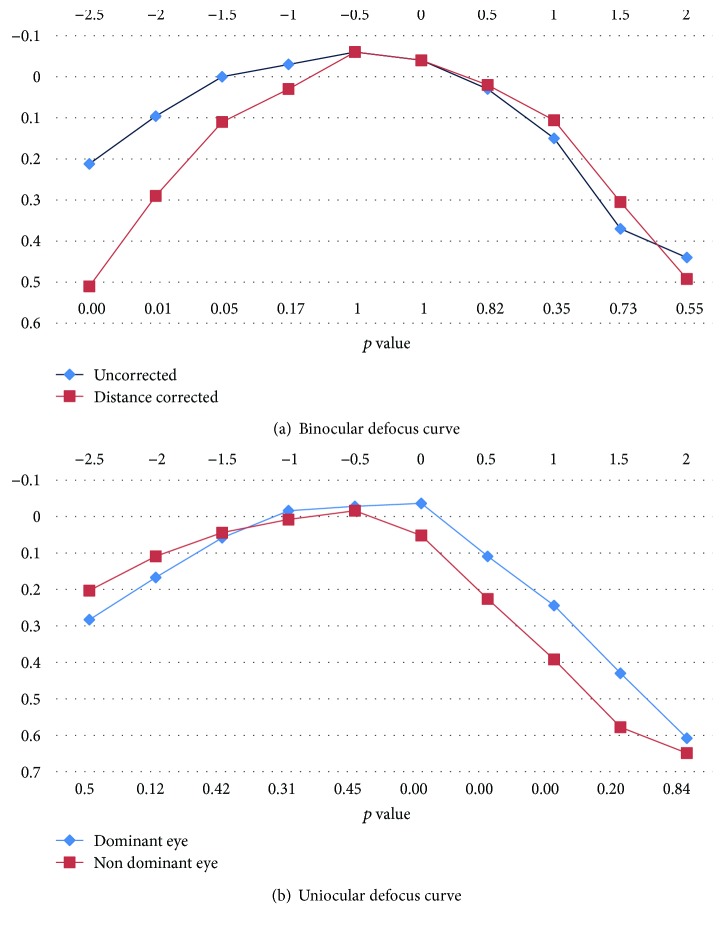
Defocus curve results from −2.50 to +2.50 D. (a) Binocular and (b) uniocular.

Figure 3(a) shows the binocular uncorrected and distance-corrected defocus curves under photopic conditions. The uncorrected defocus curve showed a visual acuity of 0 logMAR or better through the defocus range from −1.50 to 0.00 D, with a distinct peak observed at −0.50 D, consistent with the average spherical equivalent postoperative manifest refraction of −0.25 D. However, throughout the defocus range of −2.50 to +1.00 D, the visual acuity remained 0.2 logMAR or better. The distance-corrected defocus curve also showed a peak at −0.50 D, which coincided with the peak of the uncorrected defocus curve. A possible explanation for this is the need for a careful “push plus” refraction technique after Symfony implantation. It is possible that these patients were slightly “overminused” in the postop refraction. However, the uncorrected defocus curve at the defocus of −2.50, −2.00, −1.50, and −1.00 D was significantly better compared to the distance-corrected defocus curve, with no significant difference in the mean visual acuity at subsequent levels of defocus beyond −0.50 D.

We also compared the defocus curves of the dominant and nondominant eyes uniocularly at the last visit. The uncorrected vision in the nondominant eye was better than in the dominant eye from −2.50 to −1.50 D and was significantly better at −2.50 D defocus. However, beyond the defocus of −1.50 D, the dominant eye showed better UDVA which was significantly better for the defocus range of 0.00 to 1.00 D (*p* = 0.00) (Figure 3(b)). Although the nondominant eye was aimed for −0.75 D myopia, the mean UDVA in these eyes was 0.2 logMAR or better through a defocus range from −2.50 to +0.50 D. This demonstrates the functional improvement of micromonovision with the Symfony lens through a broad defocus range.

## 7. Contrast Sensitivity

**Figure 4:**
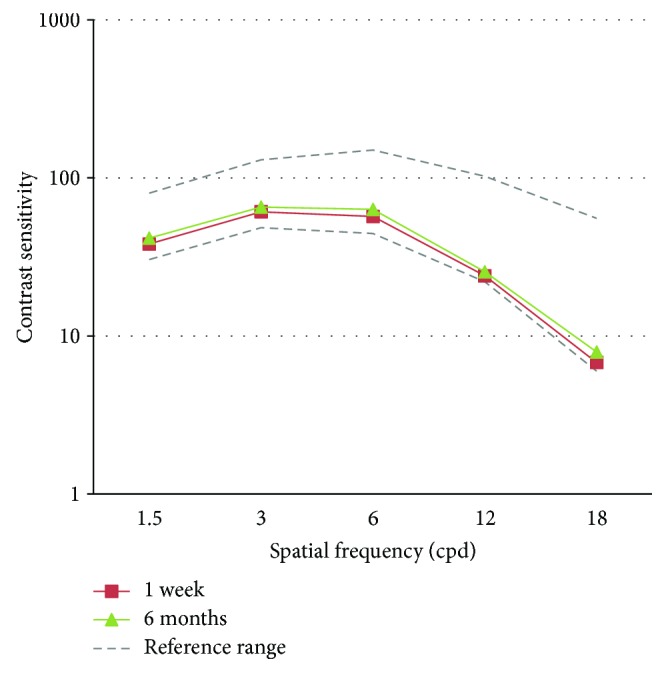
Contrast sensitivity (FACT) over time.

Figure 4 shows the binocular distance-corrected contrast sensitivity under mesopic conditions at 1 week and 6 months postoperatively. At 1 week, contrast sensitivity was within the normal range which showed improvement at 6 months. However, the change was not statistically significant for any spatial frequency (*p* > 0.05).

## 8. Dysphotopic Phenomena and Spectacle Independence Evaluation

**Figure 5:**
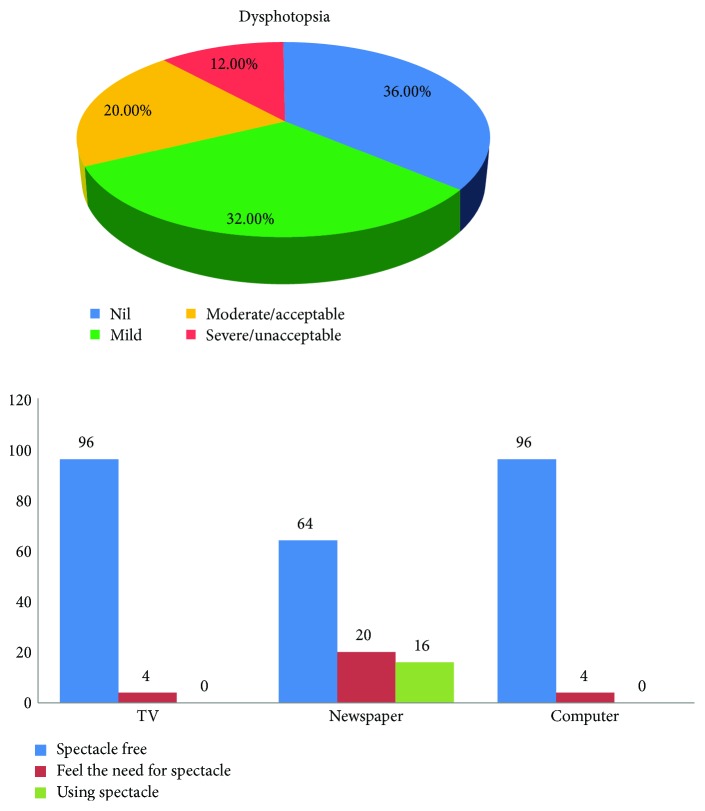
Patient satisfaction and dysphotopsia evaluation at the last visit.

At the end of 6 months, 64% (16/25) of the patients complained of dysphotopsia varying from mild to severe/unacceptable when using a directed questionnaire. For both distance- and intermediate-range activities such as watching television and computer, 96% (24/25) of the patients were highly satisfied and spectacle free. For near vision, 84% (21/25) of the subjects were completely spectacle independent with the use of ERV IOLs targeted for micromonovision. Four patients reported using glasses on occasion for reading fine print (Figure 5).

## 9. Long-Term Complications

No visually significant complications such as posterior capsular opacification, cystoid macular oedema, postop uveitis, or glaucoma occurred in any of the eyes at the end of the 6-month follow-up.

## 10. Discussion

Recently, Pedrotti et al. have compared the outcomes of Tecnis Symfony IOL with those of Tecnis monofocal IOL and concluded that the ERV IOLs provided better distance, intermediate, and near visual acuity than the aspheric monofocal IOL, while maintaining the same level of visual quality [15]. However, in their study, both eyes were targeted for emmetropia and micromonovision was not performed.

Comparing our results with the binocular visual outcomes reported by Pedrotti et al, our study showed marginally better results for mean UDVA, UNVA, UIVA (60 cm), and CDVA at the end of the 6-month follow-up (Table 6).

This shows that the treatment planning in the present study by micromonovision was in general successful in providing satisfactory outcomes through a continuous range of vision. However, their patient satisfaction evaluation method was different from the method used in our study as we evaluated the spectacle independence for specific activities at various distances.

Our results were further consistent with those of the recently published multicentric study by the CONCERTO group, in which bilateral implantation of the Symfony IOL with micromonovision provided significantly better uncorrected intermediate and near visual acuity compared to that with the nonmonovision group [16] (Table 6). However, our sample size is much smaller compared to that of the CONCERTO study. Data from a comparable number of subjects may provide better comparison of the results between the two studies.

From the results of this survey, we found that the patient satisfaction was excellent for distance- and intermediate-range activities and good for near-range activities, as 84% of the patients were completely spectacle free and only 4 patients reported using reading glasses on occasion at the end of 6 months. Detailed evaluation of this subgroup of patients revealed that there was a significant variation in the range of axial length, anterior chamber depth, and keratometry amongst the eyes included in the study (Table 7).

These factors have been shown to influence the effective lens position, which can potentially change the near point of focus of a multifocal implant [17, 18]. Savini et al. demonstrated that longer eyes with steeper corneas showed the longest near focal distance and could experience more difficulties in focusing near objects after surgery and the opposite was true for short hyperopic eyes [18]. In another study, it was proposed that caution must be exercised while planning a multifocal IOL with low add, especially in eyes with long axial lengths and deep anterior chamber depth, since the add at the spectacle plane is further expected to reduce in such cases leading to an insufficiency for near vision [16]. This may be especially relevant in the context of an ERV IOL, which would theoretically provide a lower range of addition at the IOL plane as the focus is an extended one and not a fixed point for near vision, although some authors describe the ERV IOL as the multifocal IOL with a very low add of +1.75 D, based on their results using an optical bench under monochromatic light conditions [11]. However, it was recently emphasized by Koch and Wang that using a monochromatic light as a testing parameter does not reflect real-world vision. Since the Tecnis Symfony IOL corrects for chromatic aberration, testing with the white light would rather better simulate the patients' experience [19].

The mean residual spherical equivalent in the dominant eye was −0.22 D (range = 0 to −1.50 D), and in the nondominant eye, it was −0.74 D (range = 0 to −1.25 D) at the end of 6 months (Table 5). 92% of the dominant eyes were within ±0.5 D and the rest between −0.75 and −1.50 D. Despite this, most patients did not complain about distance vision probably due to the extended depth of focus that this IOL provides, thus forgiving the errors in biometry to some extent. This was also evident from a fairly flat defocus curve obtained with this lens which has been demonstrated in numerous other studies [20].

At the end of 6 months, 32% (8/25) of the patients had complaints of seeing moderate-to-severe halos at night when using a directed questionnaire. These patients reported visualising multiple halos instead of a single halo, typically reported after implanting an ERV intraocular lens. Interestingly, these were the patients who were extremely satisfied with their near vision outcomes and whose micromonovision was deemed to be highly successful. In eyes with targeted micromonovision, the residual refractive error may induce some degree of noticeable dysphotopsia as a trade-off for a higher degree of functional near vision.

In our study, the binocular distance-corrected near and intermediate visual acuities were found to be worse compared to the uncorrected values at all postoperative visits. This was due to the reason that the targeted postoperative refractive error was slightly myopic in the dominant eyes and more myopic in the nondominant eye (due to micromonovision); hence, when corrected for distance, an expected deterioration in near and intermediate visual acuities was observed as the intended effect of micromonovision was eliminated by correcting the targeted residual refractive error that results in greater functional intermediate and near vision.

It has been reported in various studies that loss of reading ability can significantly affect the patients' quality of life [21, 22]. Hence, it is important that optimal reading ability is achieved after cataract surgery, which can be evaluated by measuring reading acuity and reading speeds which are the component aspects to one's ability to read adequately. In the present study, reading performance was assessed using the SRD, which is designed to simultaneously measure reading acuity and speed. Many studies have evaluated reading performance with various multifocal IOLs using the SRD [14, 23].

A constant improvement in the uncorrected reading acuity and reading speed was observed over time for all distances, both being highest at the end of 6 months. This can partially be attributed to the neural adaptation process and the learning curve effect that occur when patients repeat the same test again.

In conclusion, the preliminary results of our study with relatively small number of enrolled eyes suggest that micromonovision with the ERV IOL was well tolerated and led to excellent outcomes for most activities at all distances. However, further research involving a larger sample size is required to verify these results. Future studies comparing the outcomes of micromonovision with the ERV lens and low-add multifocal IOLs are suggested to evaluate their performance and patient satisfaction.

## Figures and Tables

**Table 1:** Preoperative and demographic data of all the eyes (*n* = 50) included in the study.

Parameter	Mean ± SD	Range
Age (years)	60.76 ± 10.74	41–82
Male : female	9 : 16	
UDVA (logMAR)	0.37 ± 0.32	0.10–2.00
CDVA (logMAR)	0.31 ± 0.35	0.00–2.00
SE (D)	0.92 ± 1.29	−2.00 to +4.50
*K* mean (D)	44.29 ± 1.43	41.75–46.75
Ast (D)	−0.57 ± 0.29	−1.14 to 0.00
AL (mm)	23.43 ± 0.82	22.08–25.91
ACD (mm)	3.166 ± 0.35	2.50–4.09

UDVA: uncorrected distance visual acuity; CDVA: corrected distance visual acuity; SE: spherical equivalent; Ast: astigmatism; AL: axial length; D: dioptres; ACD: anterior chamber depth.

**Table 2:** Binocular uncorrected and distance-corrected visual outcomes at 1 week, 1 month, and 6 months postoperatively.

LogMAR (mean ± SD)	1 week	1 month	6 months	*p* value^∗^
UDVA	−0.008 ± 0.09	−0.024 ± 0.14	−0.036 ± 0.09	0.18
Range	−0.20 to 0.20	−0.20 to 0.30	−0.20 to 0.10	
CDVA	−0.096 ± 0.06	−0.100 ± 0.07	−0.108 ± 0.07	0.25
Range	−0.20 to 0.00	−0.30 to 0.00	−0.20 to 0.00	
*p* value^†^	0.00	0.003	0.007	
UNVA (40 cm)	0.184 ± 0.09	0.172 ± 0.09	0.152 ± 0.11	0.05
Range	0 to 0.3	0 to 0.3	0 to 0.30	
DCNVA (40 cm)	0.232 ± 0.09	0.224 ± 0.9	0.216 ± 0.10	0.35
Range	0 to 0.4	0 to 0.3	0 to 0.4	
*p* value^†^	0.08	0.07	0.05	
UIVA (60 cm)	0.088 ± 0.09	0.068 ± 0.11	0.048 ± 0.09	0.02
Range	−0.10 to 0.3	−0.10 to 0.30	−0.20 to 0.20	
DCIVA (60 cm)	0.124 ± 0.09	0.116 ± 0.09	0.104 ± 0.08	0.13
Range	0 to 0.3	0 to 0.3	−0.1 to 0.20	
*p* value^†^	0.14	0.07	0.04	
UIVA (80 cm)	−0.016 ± 0.11	−0.032 ± 0.11	−0.044 ± 0.09	0.05
Range	−0.20 to 0.30	−0.20 to 0.20	−0.20 to 0.10	
DCIVA (80 cm)	0.016 ± 0.09	0.016 ± 0.106	0.012 ± 0.09	0.73
Range	−0.20 to 0.30	−0.20 to 0.20	−0.2 to 0.2	
*p* value^†^	0.23	0.15	0.02	

UDVA = uncorrected distance visual acuity; CDVA = corrected distance visual acuity; UNVA = uncorrected near visual acuity; DCNVA = distance-corrected near visual acuity; UIVA = uncorrected intermediate visual acuity; DCIVA = distance-corrected intermediate visual acuity. ^∗^Wilcoxon signed-rank test (comparison between 1-week and 6-month results). ^†^Mann–Whitney test.

**Table 3:** Comparison of intermediate visual performance at 60 cm and 80 cm with the ETDRS chart and Salzburg reading desk.

	Intermediated visual performance					
	Uncorrected reading acuity (UCRA)			Distance-corrected reading acuity (DCRA)		
ETDRS (logMAR)	60 cm	80 cm	*p* value	60 cm	80 cm	*p* value
1 week	0.088 ± 0.09	−0.016 ± 0.11	0.000	0.124 ± 0.09	0.016 ± 0.09	0.000
1 month	0.068 ± 0.11	−0.032 ± 0.11	0.003	0.116 ± 0.09	0.016 ± 0.106	0.001
6 months	0.048 ± 0.09	−0.44 ± 0.09	0.001	0.104 ± 0.08	0.012 ± 0.09	0.002
SRD (logMAR)						
1 week	0.114 ± 0.12	0.102 ± 0.140	0.49	0.156 ± 0.190	0.094 ± 0.16	0.62
1 month	0.102 ± 0.16	0.078 ± 0.10	0.80	0.130 ± 0.14	0.130 ± 0.15	0.32
6 months	0.094 ± 0.15	0.064 ± 0.11	0.89	0.099 ± 0.14	0.120 ± 0.15	0.90
SRD (WPM)						
1 week	110.00 ± 28.87	109.24 ± 26.72	0.82	111.00 ± 28.05	108.16 ± 23.28	0.98
1 month	112.48 ± 36.39	109.40 ± 23.62	0.61	112.48 ± 36.39	108.00 ± 21.78	0.47
6 months	119.9 ± 35.49	115.04 ± 31.56	0.59	119.9 ± 35.49	111.16 ± 25.53	0.68

*p* value using Mann–Whitney test. SRD: Salzburg reading desk; WPM: words per minute.

**Table 4:** Binocular uncorrected and distance-corrected reading acuity and reading speeds with SRD over time.

SRD (mean ± SD)	1 week	1 month	6 months	*p* value^∗^
LogMAR				
UCRA (40 cm)	0.183 ± 0.16	0.142 ± 0.15	0.132 ± 0.13	0.05
DCRA (40 cm)	0.220 ± 0.12	0.198 ± 0.12	0.188 ± 0.091	0.11
*p* value^†^	0.36	0.10	0.04	
UCRA (60 cm)	0.114 ± 0.12	0.102 ± 0.16	0.094 ± 0.15	0.37
DCRA (60 cm)	0.156 ± 0.190	0.130 ± 0.14	0.099 ± 0.14	0.06
*p* value^†^	0.60	0.26	0.71	
UCRA (80 cm)	0.102 ± 0.140	0.078 ± 0.10	0.064 ± 0.11	0.10
DCRA (80 cm)	0.094 ± 0.16	0.130 ± 0.15	0.120 ± 0.15	0.73
*p* value^†^	0.40	0.95	0.26	
Reading speed (Wpm)				
UCRS (40 cm)	115.16 ± 38.23	116.92 ± 35.05	122.84 ± 33.50	0.22
DCRS (40 cm)	110.32 ± 37.25	114.68 ± 35.19	115.24 ± 32.56	0.15
*p* value^†^	0.63	0.59	0.31	
UCRS (60 cm)	110.00 ± 28.87	112.48 ± 36.39	119.9 ± 35.49	0.42
DCRS (60 cm)	111.00 ± 28.05	112.04 ± 23.34	113.40 ± 21.22	0.49
*p* value^†^	0.94	0.61	0.93	
UCRS (80 cm)	109.24 ± 26.72	109.40 ± 23.62	115.04 ± 31.56	0.66
DCRS (80 cm)	108.16 ± 23.28	108.00 ± 21.78	111.16 ± 25.53	0.95
*p* value^†^	0.93	0.83	0.65	

UCRA = uncorrected reading acuity; DCRA = distance-corrected reading acuity; UCRS = uncorrected reading speed; DCRS = distance-uncorrected reading speed; Wpm = words per minute. ^∗^Wilcoxon signed-rank test (*p* value of 6-month result compared to 1-week result). ^†^Mann–Whitney test.

**Table 5:** Postoperative refractive outcomes of dominant and nondominant eyes over time.

Mean ± SD (dioptres)	Pre	1 week	1 month	6 months
Dominant eye				
SE	0.70 ± 1.17	−0.25 ± 0.32	−0.26 ± 0.31	−0.22 ± 0.37
Range		−1.25 to 0.00	−1.25 to 0.00	−1.25 to 0.00
*p* value		0.00	0.18	0.42
Sphere	1.00 ± 1.24	−0.180 ± 0.31	−0.180 ± 0.31	−0.07 ± 0.33
Range	−1.75 to 2.5	−1.25 to 0.00	−1.25 to 0.00	−1.25 to 0.00
*p* value		0.00	—	0.588
CYL	−0.74 ± 0.69	−0.14 ± 0.27	−0.17 ± 0.27	−0.21 ± 0.37
Range	−2.5 to 0.00	−1 to 0.00	−1 to 0.00	−1.25 to 0.00
*p* value		0.00	0.185	0.703
Nondominant eye				
SE	1.14 ± 1.38	−0.75 ± 0.37	−0.73 ± 0.46	−0.74 ± 0.44
Range		−1.5 to 0.0	−1.5 to 0.0	−1.5 to 0.0
*p* value		0.00	0.72	0.87
Sphere	1.48 ± 1.48	−0.56 ± 0.28	−0.53 ± 0.30	−0.57 ± 0.41
Range	−2 to 4.5	−1 to 0.00	−1 to 0.00	−1.5 to 0.00
*p* value		0.00	0.376	1.00
CYL	−0.66 ± 0.74	−0.39 ± 0.30	−0.41 ± 0.28	−0.38 ± 0.32
Range	−3.50 to 0.00	−1.00 to 0.00	−1.00 to 0.00	−1.00 to 0.00
*p* value		0.07	0.425	0.491

SE: spherical equivalent; CYL: cylinder. *p* values calculated using Wilcoxon signed-rank test.

**Table 6:** Comparison of binocular visual results between Pedrotti et al., CONCERTO study, and the present study.

Parameter	Pedrotti et al. (*n* = 25 patients)	CONCERTO study (monovision group) (*n* = 112 patients)	Present study (*n* = 25 patients)
UDVA			
Mean SD	0.00 ± 0.09	0.04 ± 0.11	−0.036 ± 0.09
Range	−0.20 to 0.20	0.30 to 0.40	−0.20 to 0.10
UIVA (60 cm)			
Mean SD	0.10 ± 0.09	0.09 ± 0.17	0.048 ± 0.09
Range	0.00 to 0.25	0.20 to 0.48	−0.20 to 0.20
UNVA			
Mean SD	0.18 ± 0.08	0.17 ± 0.18	0.152 ± 0.11
Range	0.00 to 0.35	0.10 to 0.70	0 to 0.30
CDVA			
Mean SD	−0.08 ± 0.07	Not evaluated	−0.108 ± 0.07
Range	−0.20 to 0.10	Not evaluated	−0.20 to 0.00
DCIVA (60 cm)			
Mean SD	0.10 ± 0.09	Not evaluated	0.104 ± 0.08
Range	0.00 to 0.25	Not evaluated	−0.1 to 0.20
DCNVA			
Mean SD	0.21 ± 0.07	Not evaluated	0.216 ± 0.10
Range	0.10 to 0.30	Not evaluated	0 to 0.4

UDVA = uncorrected distance visual acuity; CDVA = corrected distance visual acuity; UNVA = uncorrected near visual acuity; DCNVA = distance-corrected near visual acuity; UIVA = uncorrected intermediate visual acuity; DCIVA = distance-corrected intermediate visual acuity.

**Table 7:** Evaluation of axial length, anterior chamber depth, and keratometry of the eyes of the patients complaining of unsatisfactory near vision at the end of 6 months.

Sr number		Km (D)	AL (mm)	ACD (mm)	BO UNVA (logMAR)
1	RE	46.75	22.16	3.19	0.3
	LE	46.50	22.08	2.99	
2	RE	46.00	23.39	3.65	0.3
	LE	46.75	23.01	3.61	
3	RE	42.00	24.21	3.45	0.3
	LE	42.50	24.00	3.53	
4	RE	46.75	25.73	3.13	0.3
	LE	46.75	25.91	3.34	

Km: mean keratometry; D: dioptre; AL: axial length; ACD: anterior chamber depth; BO UNVA: binocular uncorrected near visual acuity.
